# Clinical and Functional Characterization of PDE1A as a Wnt/**β**-Catenin-Linked Biomarker of Progression and Platinum Resistance in Epithelial Ovarian Cancer

**DOI:** 10.32604/or.2025.072105

**Published:** 2026-02-24

**Authors:** Gwan Hee Han, Hee Yun, Joon-Yong Chung, Jae-Hoon Kim, Hanbyoul Cho

**Affiliations:** 1 Department of Obstetrics and Gynecology, Chung-Ang University College of Medicine, 84 Heukseok-ro, Dongjak-gu, Seoul, 06974, Republic of Korea; 2 Department of Obstetrics and Gynecology, Chung-Ang University Hospital, Chung-Ang University College of Medicine, 102 Heukseok-ro, Dongjak-gu, Seoul, 06973, Republic of Korea; 3 Department of Obstetrics and Gynecology, Gangnam Severance Hospital, Yonsei University College of Medicine, Gangnam-gu, Seoul, 06299, Republic of Korea; 4 Molecular Imaging Branch, Center for Cancer Research, National Cancer Institute, National Institutes of Health, Bethesda, MD 20892, USA; 5 Department of Obstetrics and Gynecology, Yonsei University College of Medicine, Seoul, 06299, Republic of Korea; 6 Institute of Women’s Life Medical Science, Yonsei University College of Medicine, Seoul, 03722, Republic of Korea

**Keywords:** Beta-catenin, epithelial ovarian cancer, phosphodiesterase 1A, wingless-related integration site (Wnt), biomarker

## Abstract

**Objectives:**

Phosphodiesterase 1A (PDE1A) regulates intracellular cyclic nucleotide signaling and has been implicated in tumor progression, but its clinical relevance and functional role in epithelial ovarian cancer (EOC), particularly in relation to the response to platinum remain unclear. This study aimed to evaluate the clinical significance of PDE1A in EOG and to clarify its functional role in tumor progression and response to platinum-based chemotherapy.

**Methods:**

PDE1A mRNA and protein levels were analyzed using public databases, RNA sequencing, and immunohistochemistry. Correlations between PDE1A expression, clinicopathological features, and prognosis were assessed. Functional roles were investigated in ovarian cancer cell lines.

**Results:**

PDE1A was significantly overexpressed in EOC tissues compared with that in normal ovarian epithelial tissues. Overexpression correlated with advanced International Federation of Gynecology and Obstetrics (FIGO) stage, poor tumor grade, and reduced response to platinum-based chemotherapy. High PDE1A levels were linked to worse disease-free survival and overall survival, and multivariate analysis confirmed PDE1A as an independent prognostic factor. To elucidate its functional role, we performed *in vitro* experiments showing that PDE1A knockdown suppressed cell proliferation and colony formation, induced G1 arrest, and downregulated β-catenin signaling with reduced cyclin D1 and c-Myc expression. Notably, these inhibitory effects were partially rescued by lithium chloride (LiCl), a Wingless-related integration site (Wnt)/β-catenin activator.

**Conclusions:**

In conclusion, our findings identify PDE1A as a Wnt/β-catenin–linked biomarker of tumor progression and platinum resistance in EOC and provide a biological rationale for further investigation of PDE1A-targeted strategies in preclinical models.

## 1 Introduction

Epithelial ovarian cancer (EOC) remains one of the most lethal gynecologic malignancies worldwide. Recent global estimates indicate that approximately 324,000 new ovarian cancer cases and 206,839 deaths occurred in 2022 [1]. Because early-stage disease is often asymptomatic and reliable screening tools are lacking, the majority of patients are still diagnosed at an advanced stage, when the 5-year survival rate drops below 30% despite aggressive treatment [2,3]. Standard management comprises cytoreductive surgery followed by platinum-taxane combination chemotherapy. While initial response rates approach 80%, durable remission is uncommon: 20%–30% of patients develop progressive or recurrent disease within 6 months of completing first-line therapy, and up to 75% of advanced-stage cases ultimately relapse [2,3]. The current therapeutic strategies for EOC predominantly include debulking surgery and platinum-based chemotherapy. Nonetheless, despite the initial responsiveness to platinum-based chemotherapy in approximately 80% of patients with EOC, the long-term efficacy of these therapies is significantly hampered by a high recurrence rate. Within six months of chemotherapy, approximately 20%–30% of patients experience relapse or disease progression, and ca. 75% of women with advanced EOC face recurrence that is often incurable [2]. These outcomes emphasize the need to elucidate mechanisms driving EOC progression and treatment resistance and to identify biomarkers for risk stratification and therapeutic selection.

Molecular studies of EOC have highlighted dysregulated Ca^2+^/calmodulin (CaM)–dependent signaling as a promising area of investigation. CaM, a ubiquitous Ca^2+^-binding protein, regulates many Ca^2+^-dependent processes, including proliferation and apoptosis, and aberrant Ca^2+^/CaM pathways have been implicated in tumor development [4]. Cyclic nucleotide phosphodiesterases (PDEs), a key group of CaM-regulated effectors, hydrolyze cyclic adenosine monophosphate (cAMP) and cyclic guanosine monophosphate (cGMP) and thereby shape compartmentalized second-messenger signaling [5]. Across different cancers, including breast cancer and hepatocellular carcinoma (HCC), altered expression or activity of several PDE families has been reported to lower intracellular cAMP and cGMP and promote proliferation and survival. In colon cancer, pharmacologic PDE inhibitors can restore cyclic nucleotide signaling and suppress tumor growth [6]. In EOC, dysregulation of several PDE isoforms, including members of the PDE3, PDE4, PDE5, and PDE10 families, has been reported, and in experimental models, modulation of cAMP/cGMP pathways has been linked to changes in proliferation and chemosensitivity [7–10]. In particular, cGMP-elevating strategies, such as treatment with cGMP analogues or PDE inhibition, have been shown to inhibit tumor progression, supporting a tumor-suppressive role for cGMP–protein kinase G (PKG) signaling in EOC [6]

Among the 11 PDE families, the PDE1 subfamily comprises Ca^2+^/CaM-activated enzymes with distinct substrate specificities and tissue distributions. PDE1A preferentially hydrolyzes cGMP over cAMP, whereas PDE1C degrades both cyclic nucleotides with similar efficiency [6,11]. Experimental studies in other malignancies have implicated PDE1 isoforms in tumor progression, and in several preclinical models, PDE1 inhibition has shown antitumor activity by increasing cGMP and modulating downstream pathways such as PKG and Wnt/β-catenin signaling pathway [12]. Our bulk RNA sequencing cohort and public gene expression datasets (GSE14001 and GSE66957) revealed higher PDE1A expression in EOC than in non-adjacent normal ovarian epithelial tissues [13,14]. Based on its known biochemical preference for cGMP, its Ca^2+^/CaM-dependent regulation, and its upregulation in these datasets, we focused on PDE1A as a Ca^2+^/CaM-regulated phosphodiesterase that might contribute to cyclic nucleotide remodeling in EOC.

Therefore, in this study, we aimed to characterize the expression pattern and clinical significance of PDE1A in EOC and to determine whether PDE1A promotes EOC progression, at least in part, through β-catenin-related signaling.

## 2 Materials and Methods

### 2.1 Patients and Tumor Specimens

Formalin-fixed, paraffin-embedded (FFPE) ovarian tissues were retrospectively collected from women who underwent primary cytoreductive surgery for adnexal masses at Gangnam Severance Hospital (Seoul, Republic of Korea) between 1996 and 2021. The series comprised 173 epithelial ovarian cancer (EOC) cases, 60 borderline ovarian tumors, 81 benign epithelial lesions, and 80 tumour-distant normal ovarian surface epithelia. Normal controls were obtained from grossly uninvolved ovarian cortex that was located at least 2 cm away from the tumour margin; whenever possible, tissue was sampled from the contralateral ovary. All normal specimens were reviewed on haematoxylin and eosin (H&E)-stained slides by gynecologic pathologists to exclude cytologic atypia or dysplasia, and cores for tissue microarrays (TMAs) were taken from representative epithelial areas to minimize stromal or inflammatory contamination. For the EOC cohort, we included only patients who had pathologically confirmed epithelial ovarian carcinoma, underwent extensive primary debulking surgery without neoadjuvant chemotherapy or interval debulking, and received standard paclitaxel–carboplatin chemotherapy post-operatively. Patients were excluded if they had non-epithelial ovarian malignancies, palliative surgery only, insufficient FFPE material for TMA construction, or incomplete clinicopathological or follow-up data. Some cases were supplemented by specimens from the Korean Gynecologic Oncology Bank, supported by the National Research Foundation of Korea. Clinical variables, including age, FIGO 2014 stage, World Health Organization (WHO) histologic subtype and grade, treatment details, and outcomes, were abstracted from electronic medical records and re-coded to ensure uniform staging and histotyping. Disease-free survival (DFS) and overall survival (OS) were calculated from the date of primary surgery to the date of recurrence, death, or last follow-up. Tumour response was evaluated according to the Response Evaluation Criteria in Solid Tumors (RECIST) version 1.1 [15]. Platinum status was defined by the platinum-free interval (PFI): patients with PFI < 6 months were categorized as platinum-resistant or -refractory (PRR), whereas those with PFI ≥ 6 months were considered platinum-sensitive. All patients provided written informed consent for the use of their tissues and clinical data, and the study protocol was approved by the Institutional Review Board of Gangnam Severance Hospital (IRB No. 3-2024-0049), in accordance with the Declaration of Helsinki. he epithelial ovarian cancer cohort and corresponding clinicopathological data were derived from the same institutional tissue microarray previously used in our ATP6V1B1 study [14]. For the present work, serial sections from this TMA were newly stained and analysed for PDE1A expression.

### 2.2 Public Database

To corroborate our RNA sequencing data, we utilized two well-documented datasets from Gene Expression Omnibus (GEO) platform, specifically GSE14001 and GSE66957 [13]. These datasets, selected for their relevance, include comparative analyses of gene expression between normal ovarian epithelial tissues and high-grade serous ovarian cancer (HGSOC) samples, directly aligning with the focus of our research. Access to these resources was achieved through the GEO web portal (https://www.ncbi.nlm.nih.gov/geo/) in March 2023. Our search strategy was finely tuned to filter datasets using key terms such as “*Homo sapiens*,” “epithelial ovarian cancer,” and “normal ovarian epithelium,” while the technological platforms of choice for these datasets were primarily the Affymetrix microarray (Thermo Fisher Scientific; Waltham, MA, USA) and Illumina platforms (Illumina, San Diego, CA, USA) used by various research groups.

### 2.3 Laser-Capture Microdissection, RNA Extraction, and RNA Sequencing

Formalin-fixed, paraffin-embedded (FFPE) ovarian tissue samples were microdissected using the AS LMD system (Leica Microsystems, Wetzlar, Germany) after hematoxylin and eosin staining, under the supervision of an experienced pathologist to ensure histological accuracy. RNA extraction and sequencing were performed as described in our prior study on epithelial ovarian cancer 26. Briefly, total RNA was isolated using TRIzol reagent (Cat. No. 15596026; Invitrogen; Thermo Fisher Scientific), and RNA quality was assessed using a NanoDrop spectrophotometer and an Agilent 2100 Bioanalyzer (Agilent Technologies, Santa Clara, CA, USA). Because the material consisted of archival FFPE specimens, the Bioanalyzer electropherograms showed substantial RNA fragmentation and low RNA integrity, which is typical for formalin-fixed tissues; therefore, we did not use an absolute RIN or DV200 cut-off as the primary inclusion criterion. Instead, we used a 3^′^ mRNA-Seq library preparation protocol (QuantSeq 3^′^ mRNA-Seq Library Prep Kit, Lexogen, Vienna, Austria) optimized for FFPE-derived RNA and restricted the analysis to libraries that fulfilled prespecified sequencing based quality criteria (processed reads ≥ 7.5 million, uniquely mapped reads ≥ 65%, average Q20 ≥ 95% after trimming, rRNA ≤ 15%, and appropriate 3^′^ gene-body coverage for QuantSeq 3^′^. These thresholds were established based on the manufacturer’s recommendations for the QuantSeq protocol and were empirically optimized to ensure robust gene quantification despite the fragmented nature of FFPE-derived RNA.). Only libraries passing all quality control metrics were included in downstream analyses. Libraries were sequenced on an Illumina NextSeq 500 platform (Illumina) to generate 75-bp single-end reads. On average, 11.5 million reads were generated per sample (range: 8.0–15.0 million), providing adequate sequencing depth for differential expression profiling.

### 2.4 Bioinformatic Processing and Differential Gene Expression Analysis

Adaptor trimming and low-quality read removal were performed using Bbduk (BBMap v36.59). Specific trimming parameters included a minimum quality score (trimq) of 20, removal of poly-A tails and adapter sequences (ktrim = r), and a minimum read length (minlen) of 20 bp. Read quality was assessed with FastQC (v0.11.7), and samples failing to meet the QC criteria described above were excluded from the analysis [16,17]. Clean reads were aligned to the GRCh38 reference genome using the STAR aligner (v2.7.3a) [18], and gene-level counts were generated with HTSeq-count (v0.12.4) in union mode [19]. Differential gene expression analysis was conducted using the DESeq2 package (v1.26.0) in R 4.2.0 [20], with significance thresholds set at an adjusted *p*-value < 0.05 (Benjamini–Hochberg method) and an absolute log_2_ fold change > 1.

### 2.5 Tissue Microarray and Immunohistochemistry

Tissue microarrays (TMAs) were constructed by extracting 1.0 mm diameter cores from donor FFPE tissue blocks that were rich in tumor cells, using a precision tissue array system from Beecher Instruments (Silver Spring, MD, USA). The derived TMA blocks were sectioned into 5 µm slices using a rotary microtome. For immunohistochemistry staining, sections were first deparaffinized in xylene, then progressively rehydrated through an ethanol gradient to distilled water. Antigen retrieval was conducted using a high-pressure Dako Pascal system pressure cooker (model S2800; Agilent Technologies), immersing the sections in a pH 6.0 antigen retrieval buffer (cat. no. S1699; Dako; Agilent Technologies) and heating to 125°C for 2 min under 20–23 psi. The sections were then neutralized with 3% H_2_O_2_ in methanol for 10 min at room temperature to block endogenous peroxidase activity. The immunostaining protocol involved treating the sections with an anti-PDE1A rabbit polyclonal anti-body (cat. no. HPA022151; Sigma-Aldrich, St Louis, MO, USA) diluted 1:100, at room temperature for 30 min. Visualization was achieved using the Envision+ Dual Link System-HRP (Dako; Agilent Technologies Inc.) and DAB+ (3,3^′^-diaminobenzidine; Dako; Agilent Technologies) for 10 min at room temperature, followed by counterstaining with hematoxylin. The slides were finally preserved using Faramount Aqueous Mounting Medium (Dako; Agilent Technologies). For the controls, the primary antibody was excluded and IgG was used instead; validated positive controls were included in the array.

### 2.6 Evaluation of Immunohistochemistry Staining

Digital images of the stained sections were captured using a NanoZoomer 2.0 HT microscope (Hamamatsu Photonics K.K., Hamamatsu City, Japan) at ×20 magnification, providing 0.5 µm resolution. Analysis of these images was performed using Visiopharm (v6.5.0.2303; Visiopharm, Hør-sholm, Denmark), using an algorithm designed to quantify the cytoplasmic staining intensity on a scale of 0–3 and the percentage of positive cells (0%–100%). The final histoscore was computed by multiplying the intensity by the percentage of positively stained cells, offering a potential range from 0–300.

### 2.7 Cell Culture and Reagent

Human ovarian cancer cells, including OVCAR3 (ATCC^®^ HTB-161^™^), SKOV3 (ATCC^®^ HTB-77^™^), and TOV112D (ATCC^®^ CRL-11731^™^) were sourced from the American Type Culture Collection (ATCC, Manassas, VA, USA). Additional cell lines, OVCA429 (RRID:CVCL_3936) and OVCA433 (RRID:CVCL_0475) were procured from Dr. Samuel C. Mok (The University of Texas MD Anderson Center, Houston, TX). OVCA429 and OVCA433 cell lines were established cell lines derived from patients with late-stage serous ovarian adenocarcinomas, as described by Bast et al. [21]. These cells were cultured in Dulbecco’s Modified Eagle Medium (DMEM; Corning^®^ 10-013-CV; Corning, Manassas, VA, USA) supplemented with 10% fetal bovine serum (FBS; Gibco^™^ A5669701; Gibco, Life Technologies, Grand Island, NY, USA) and 1% penicillin/streptomycin (Gibco^™^ 15140122). The immortalized HOSE cell lines (iHOSE 1481 (RRID:CVCL_ZL32), 4138 (RRID:CVCL_ZL33), and 8695 (RRID:CVCL_ZL34)) had been established by introducing the viral oncoproteins HPV E6/E7 and SV40 T antigen into short-cultured human ovarian surface epithelial cells [22] were similarly maintained in DMEM with 10% FBS and 50 μg/mL gentamicin (Gibco^™^ 15750060), all incubated at 37°C in a humidified atmosphere containing 5% CO_2_. All cultures were checked for Mycoplasma contamination before experimental use (e-Myco^™^ Mycoplasma PCR Detection kit; Cat. No. 25235, iNtRON Biotechnology, Seongnam, Korea) and authenticated using short tandem repeat sequencing (IDEXX Bio Research). Chemical agents, including LiCl (Cat. No. L9650) and MG132 (Cat. No. S2619) were obtained from Sigma Aldrich (St. Louis, MO, USA) and Selleck Chemicals (Houston, TX, USA), respectively.

### 2.8 siRNA Transfection

For gene silencing, non-targeting control siRNA and specific siRNAs targeting PDE1A were acquired from Bioneer (Daejeon, Republic of Korea). The sequences for the PDE1A siRNAs were as follows: PDE1A #1 5^′^-CAUCAUUCAGCAGAACAAA-3^′^ (sense), 5^′^-UUUGUUCUGCUGAAUGAUG-3^′^ (antisense); and PDE1A #2 5^′^-GUGGAAAGAAUGUACCGAA-3^′^ (sense), 5^′^-UUCGGUACAUUCUUUCCAC-3^′^ (antisense). Transfection was performed using Lipofectamine RNAiMAX Reagent (Cat. No. 13778150; Invitrogen; Thermo Fisher Scientific) in 6-well plates using 100 pmol of siRNA per well, following the manufacturer’s guidelines.

### 2.9 Real-Time Quantitative RT-PCR

To evaluate gene expression, total RNA was extracted from various human ovarian cancer cell lines using AccuPrep^®^ Universal RNA Extraction Kit (Cat. No. K-3141; Bioneer), followed by reverse transcription using AccuPower^®^ RocketScript^™^ RT PreMix (Cat. No. K-2105; Bioneer), according to the manufacturer’s instructions. Quantitative real-time PCR was then conducted using TOPrealTM qPCR 2X PreMIX (SYBR Green with high ROX; Cat. No. RT-501S; Enzynomics, Daejeon, Republic of Korea) on an Applied Biosystems 7300 system (Applied Biosystems, Foster City, CA, USA). The PCR reaction consisted of 1 DNA denaturation step (95°C, 3 min) and 40 cycles of amplification step (95°C, 15 s; 60°C, 1 min). The dissociation curve was also generated for every run to validate the specificity of amplification. Primers were purchased from Bioneer; PDE1A 5^′^-TAGCTGCACAAGAAGCAAGAACCAG-3^′^ (forward) and 5^′^-GCTGCCACCATGCACGAGGTTT-3^′^ (reverse); CTNNB1 5^′^-GCTTTCAGTTGAGCTGACCA-3^′^ (forward) and 5^′^-CAAGTCCAAGATCAGCAGTCTC-3^′^ (reverse); GAPDH 5^′^-AGAAGGCTGGGGCTCATTTG-3^′^ (forward) and 5^′^-AGGGGCCATCCACAGTCTTC-3^′^ (reverse). Gene expression quantification was executed using the relative quantification method (2^−∆∆Ct^), where data were normalized to GAPDH expression. All procedures were rigorously validated by conducting each experimental setup in triplicate and replicating the entire process three times to affirm the consistency and reliability of the findings.

### 2.10 Cell Proliferation Assay

Cell proliferation was assessed using an EZ-Cytox cell viability assay kit (CCK-8–type; EZ-1000, Daeil Lab Service, Seoul, Republic of Korea) according to the manufacturer’s instructions. OVCA429 and OVCA433 cells were seeded into 96-well plates at a density of 8 × 10^3^ cells per well. At the indicated time points, 10 µL of EZ-Cytox reagent reagent was added to each well and the plates were incubated at 37°C for 3 h. Absorbance at 450 nm was measured using a SpectraMax^®^ ABS microplate reader (Molecular Devices, San José, CA, USA). Background absorbance from wells containing culture medium and EZ-Cytox reagent without cells was subtracted from all measurements. Each condition was tested in triplicate in three independent experiments, and data are presented as mean ± SD.

### 2.11 Colony Formation Assay

The ability of OVCA429 and OVCA433 cells to form colonies was evaluated post-transfection with siRNA targeting PDE1A. Following 48 h of siRNA exposure, 500 cells per well were seeded into 6-well plates and cultured at 37°C with 5% CO_2_. After 2 weeks, colonies were fixed with methanol for 10 min, stained with 0.5% crystal violet (Cat. No. C0775; Sigma-Aldrich) for 20 min, and the number of colonies was quantified under Zeiss Primovert microscope (Carl Zeiss, Jena, Germany), with a colony defined as a cluster consisting of more than 50 cells. This procedure was replicated three times to ensure consistency of results.

### 2.12 Cell Cycle Analysis Using Flow Cytometry

For cell cycle analysis, OVCA429 and OVCA433 cells (1 × 10^6^) were collected and fixed in 70% ethanol. Fixed cells were centrifuged, washed, and resuspended in PBS (pH 7.4) containing RNase A (100 µg/mL), followed by incubation for 30 min at room temperature. The cellular DNA was then stained with propidium iodide (PI, 50 µg/mL) for 30 min in the dark. Flow cytometric analysis was conducted using a FACSCanto II (BD Biosciences, San Jose, CA, USA), and the results were analyzed using FlowJo software version 10.8.0 (FlowJo LLC, Ashland, OR, USA).

### 2.13 Boyden Chamber Assay

The migratory and invasive potential of ovarian cancer cells was quantified using 48-well chemotaxis chambers (Neuro Probe, Gaithersburg, MD, USA) equipped with polycarbonate membranes with 8-µm pores (PFB8; Neuro Probe). For the invasion assay, membranes were pre-coated with Matrigel matrix (Cat. No. 354230; Corning, Corning, NY, USA) diluted in serum-free medium to a final concentration of 300 µg/mL in a total volume of 3 mL and allowed to polymerize at 37°C. For the migration assay, the same procedure was performed without Matrigel coating. The lower wells were filled with DMEM supplemented with 10% FBS as a chemoattractant, while the upper wells were seeded with 1 × 10^5^ siRNA-transfected cells suspended in 50 μl of medium containing 0.05% FBS. After 24 h of incubation at 37°C in 5% CO_2_, non-migrated or non-invaded cells on the upper surface of the membranes were gently removed with cotton swabs, and the cells that had migrated to the lower surface were fixed in methanol and stained with Diff-Quik solution (Cat. No. 38721; Sysmex, Kobe, Japan). Cells on the lower surface of the membrane were counted under an Axio Imager M2 microscope (Carl Zeiss, Thornwood, NY, USA) in six randomly selected fields per membrane. All conditions were assessed in triplicate, and the experiment was independently repeated three times to ensure reproducibility.

### 2.14 Western Blot Analysis

Protein expression in ovarian cancer cells was analyzed post-treatment. Cells were lysed using the Cell Lysis Buffer (#9803; Cell Signaling Technology, Danvers, MA, USA) supplemented with phenylme-thylsulfonyl fluoride. Protein concentrations were measured using the Bradford Assay (Protein Assay Dye Reagent Concentrate; Cat. No. 5000006; Bio-Rad Laboratories, Inc., Hercules, CA) Proteins extracted from the lysates (20 µg per sample) were resolved on 10% (Slug and Cyclin D1) or 8% SDS-PAGE gels and transferred onto nitrocellulose membranes. The membranes were then blocked with 2% bovine serum albumin (BSA) (#9998S; Cell Signaling Technology) in TBST (TBS containing 0.1% Tween-20) for 1 h at room temperature. After blocking, the membranes were then incubated in TBST containing 2% BSA, overnight at 4°C with primary antibodies at the following dilutions: E-cadherin (CST #3195), Slug (CST #9585), Cyclin D1 (CST #2978), β-catenin (CST #8482), P-β-catenin (Ser33/37/Thr41, CST #9561), and c-Myc (sc-40; Santa Cruz, CA, USA) 1:1000; vimentin (CST #5741) and α-actinin (sc-17829) 1:5000. Following primary antibody incubation, the membranes were incubated with HRP-conjugated secondary antibodies diluted in TBST. Anti-rabbit IgG, HRP-linked Antibody (CST #7074S) and anti-mouse IgG, HRP-linked Antibody (CST #7076S) were used at a 1:2000 dilution and incubated for 1 h at room temperature. The membranes were developed using enhanced chemiluminescence (Cat. No. 34580; Thermo Fisher Scientific) to visualize the bands.

### 2.15 Cyclic GMP Assays

Intracellular cGMP content was measured using the Cyclic GMP XP Assay Kit (#4360; Cell Signaling Technology, Danvers, MA, USA). Briefly, 4 × 10^4^ cells per well were seeded in 96-well plates and transfected with siRNA (1 pmol/well) using Lipofectamine RNAiMAX (Thermo Fisher Scientific) according to the manufacturer’s protocol. After 48 h of transfection, the cells were lysed with 100 μL of lysis buffer provided in the kit. Following cell lysis, intra-cellular cGMP was quantified by ELISA microplate reader (SpectraMax^®^ ABS, Molecular Devices, San José, CA, USA) according to the manufacturer’s instructions.

### 2.16 Statistical Analysis

Statistical analyses were performed utilizing SPSS 25.0 (IBM Corp., Armonk, NY, USA) and R 4.2.0. The expression of PDE1A and its association with clinicopathological variables in EOC were assessed using the Mann–Whitney *U* or Kruskal–Wallis test, as appropriate. Survival outcomes were analyzed using the Kaplan–Meier method with log-rank tests to compare curves for survival analysis, and multivariate survival analysis was conducted using a Cox proportional hazards model. An optimal H-score cut-off for PDE1A expression was determined using the “MaxStat” package in R, and data were stratified accordingly [23]. The candidate cut points were limited to the 10th to 90th percentiles of the PDE1A H-score distribution, and each group was required to include at least 15% of cases (minprop = 0.15). The optimal cut-off was determined using DFS as the primary endpoint, and the same threshold was subsequently applied to the analysis of OS. To address multiplicity in the MaxStat search, we used conditional Monte Carlo adjustment with 10,000 replicates. To assess potential overfitting, we performed stratified 10-fold cross-validation and a 0.632+ bootstrap with 200 resamples. All tests were two-sided, and effects with *p* < 0.05 was considered statistically significant.

## 3 Results

### 3.1 PDE1A is Upregulated in EOC Samples, and Its Overexpression Correlates with Unfavorable Survival of Patients with EOC

**Figure 1:**
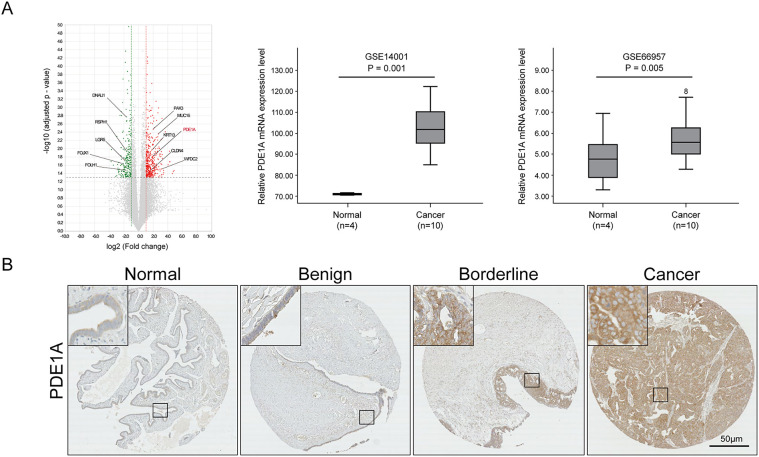

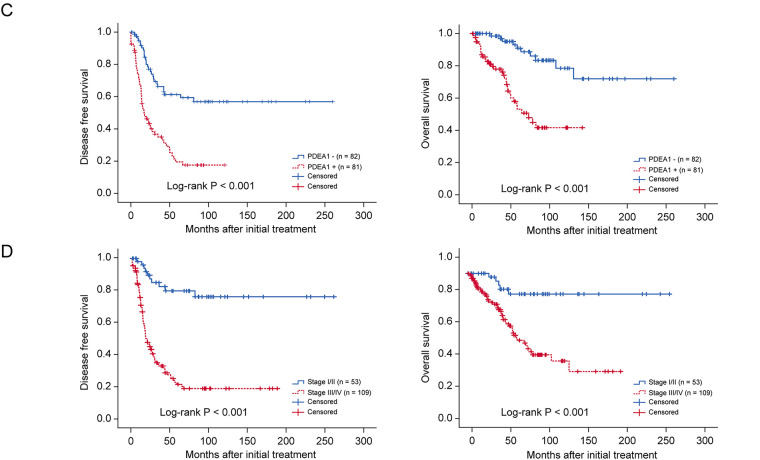
Comparative analysis of PDE1A expression in epithelial ovarian cancer. (**A**) Volcano plot showing differentially expressed genes between normal ovarian epithelial tissues and epithelial ovarian cancer (EOC) tissues based on RNA-sequencing analysis. The *x*-axis represents log_2_ (fold change, cancer vs. normal) and the *y*-axis represents −log _10_ (adjusted *p*-value). Significantly deregulated genes (red, upregulated; green, downregulated) were defined by adjusted *p* < 0.05 and |log_2_ (fold change) | > 1 (i.e., ≧2-fold change). Vertical dotted lines at log_2_ (fold change) = ±1 and the horizontal dotted line at −log_10_ (0.05) indicate the fold-change and significance thresholds, respectively. PDE1A (highlighted in red) is among the significantly upregulated genes. Plots were generated using ExDEGA v1.6.5 software (Ebiogen, Seoul, Republic of Korea). PDE1A is highlighted in red, and the top upregulated (MUC16, WFDC2, CLDN4, KRT13, PAX3) and downregulated (DNALI1, RSPH1, LGR5, FOXJ1, FOLH1) genes are annotated. Box plots for the mRNA expression of PDE1A between normal and high-grade serous ovarian cancer samples. Publicly available gene expression data were obtained from the gene expression omnibus (GEO) database (GSE14001 and GSE66957). Boxes represent the interquartile range (IQR), horizontal lines indicate the median, whiskers denote the range within 1.5× IQR, and open circles with numbers represent individual outlier samples whose expression values lie beyond the whisker range. (**B**) Representative images of immunohistochemical staining of PDE1A in non-adjacent ovarian epithelial tissues (normal), benign and borderline tumor, and EOC samples. (Main: 50 μm, Insets: 10 μm scale) (**C**) Kaplan–Meier survival curves for disease-free survival and overall survival according to PDE1A expression in patients with EOC. Groups: PDE1A+ (*H*-score > 83.9) vs. PDE1A− (*H*-score ≤ 83.9); cut-off determined by MaxStat (log-rank; conditional Monte Carlo adjustment; minprop = 0.15). (**D**) Disease-free survival and overall survival in patients with EOC according to International Federation of Gynecology and Obstetrics stage

We first investigated PDE1A expression in EOC samples. Bulk RNA sequencing revealed significant PDE1A mRNA upregulation in our EOC tissue samples relative to normal ovarian tissue, a finding we corroborated using publicly available datasets (GSE14001 and GSE66957) (Fig. 1A) [13]. In addition to PDE1A, the top upregulated genes (MUC16, WFDC2, CLDN4, KRT13, and PAX3) and top downregulated genes (DNALT1, RSPH1, LGR5, FOXJ1, and FOLH1) are annotated in Fig. 1A to highlight the key transcriptomic differences between normal and malignant tissue. At the protein level, IHC of TMAs including 159 EOCs, 43 borderline tumors, 77 benign tissues, and 72 non-adjacent normal ovarian tissue samples confirmed upregulation of PDE1A expression in EOCs relative to non-adjacent normal ovarian epithelial tissue, benign ovarian tumors, and borderline tumors (*p* < 0.001, Fig. 1B and Table 1). PDE1A was predominantly localized in the cytoplasm of tumor cells.

**Table 1:** Expression of PDE1A in relation to clinicopathological characteristics in immunohistochemical analysis

	No.	%	Mean score (95% CI)	*p*-Value
**All study subjects**	351	100		
**Diagnostic category**				
Normal	72	20.5	14.63 [10.34–18.92]	<0.001
Benign	77	21.9	28.81 [23.85–35.77]	
Borderline	43	12.3	36.53 [28.09–44.98]	
Cancer	159	45.3	49.12 [43.14–55.10]	
**FIGO stage**				
I–II	50	31.4	38.98 [29.47–48.48]	0.025
III–IV	109	68.6	53.67 [46.12–61.22]	
**Cell type**				
Serous	106	66.7	56.01 [48.41–63.62]	0.001
Others	53	33.3	35.21 [26.57–43.86]	
**Tumor grade**				
Well/Moderate	74	49.7	41.60 [34.54–48.68]	0.009
Poor	75	50.3	58.05 [47.94–68.16]	
**CA125**				
Negative	23	14.6	44.06 [28.69–59.43]	0.547
Positive	134	85.4	49.31 [42.66–55.95]	
**Chemosensitivity**				
Sensitive	133	92.3	48.51 [41.95–55.07]	0.014
Resistant	12	8.3	77.12 [54.96–99.29]	

Note: FIGO, International Federation of Gynecology and Obstetrics; CI, confidence interval. Percentages for FIGO stage, cell type, tumor grade, CA125, and chemosensitivity are calculated among patients with epithelial ovarian cancer (*n* = 159) and are based on the number of cases with available data for each variable (i.e., excluding missing values).

Clinically, the upregulation of PDE1A expression was significantly associated with advanced FIGO stage, higher tumor grade, serous histology and platinum resistance (*p* = 0.025, *p* = 0.001, *p* = 0.009, and *p* = 0.014, respectively) (Table 1). Using a MaxStat algorithm based on the log-rank statistic, we identified an optimal PDE1A *H*-score cut-off of 83.9. Patients were classified into a PDE1A^−^ (*H*-score ≤ 83.9) and a PDE1A^+^ (*H-*score > 83.9); the overexpression group showed significantly shorter DFS and OS (both *p* < 0.001; Fig. 1C). As expected, FIGO stage III/IV was significantly associated with poor DFS and OS (both *p* < 0.001; Fig. 1D); further, this is consistent with published findings, thus reinforcing the reliability of our cohort. The prognostic significance of PDE1A expression was further confirmed via multivariable Cox regression analysis, which identified it as an independent predictor of both DFS (hazard ratio [HR] = 3.11, 95% CI 1.85–5.24, *p* < 0.001) and OS (HR = 5.99, 95% CI 3.07–11.67, *p* < 0.001) (Table 2). Together, these findings establish PDE1A as a clinically relevant biomarker associated with aggressive tumor behavior and poor prognosis in EOC.

**Table 2:** Univariate and multivariate analyses of the association between prognostic variables and disease-free survival in epithelial ovarian cancer.

	Disease-free survival hazard ratio [95% CI], *p*-Value		Overall survival hazard ratio [95% CI], *p*-Value	
	Univariate	Multivariate	Univariate	Multivariate
Age (>50)	1.58 [1.06–2.35], 0.024	1.20 [0.75–1.90], 0.446	2.12 [1.17–3.84], 0.013	2.02 [1.01–4.02], 0.045
FIGO stage (III–IV)	6.42 [3.33–12.39], <0.001	3.78 [1.70–8.42], <0.001	5.10 [2.02–12.86], 0.001	2.58 [0.89–7.50], 0.081
Cell type (serous)	0.33 [0.20–0.55], <0.001	0.49 [0.27–0.91], 0.024	0.22 [0.09–0.56], 0.001	0.34 [0.12–0.97], 0.043
Tumor grade (poor)	1.95 [1.28–2.97], 0.002	1.36 [0.86–2.17], 0.192	1.69 [0.95–3.00], 0.076	NA
CA125^+^ (>35 U/mL)	2.39 [1.20–4.74], 0.013	0.87 [0.37–2.03], 0.751	2.22 [0.80–6.16], 0.127	NA
PDE1A^+a^	4.36 [2.69–7.07], <0.001	3.11 [1.85–5.24], <0.001	6.88 [3.67–12.88], <0.001	5.99 [3.07–11.67], <0.001

Note: NA, not applicable; ^a^cut-off value of PDE1A^+^ is over 83.9 of the IHC score; Clinicopathological variables and survival data were obtained from the shared institutional TMA cohort described in our previous ATP6V1B1 report [14]; Cox regression analyses were performed in patients with complete data for all covariates.

### 3.2 PDE1A Knockdown Inhibits Ovarian Cancer Cell Proliferation by Impeding Cell Cycle Progression

**Figure 2:**
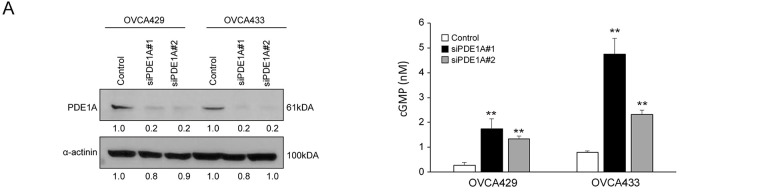

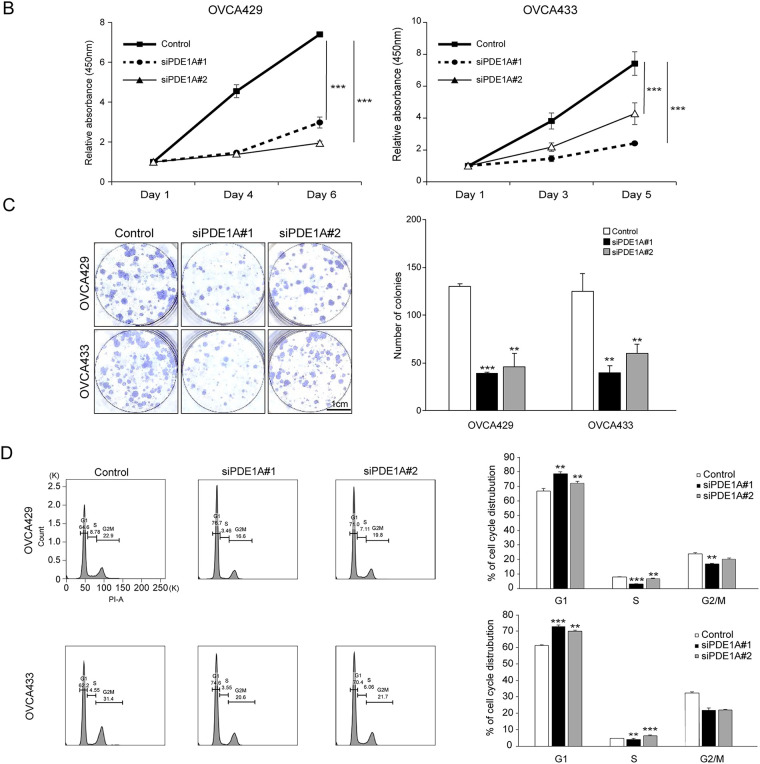
Knockdown of PDE1A expression inhibits the proliferation of ovarian cancer cells by impeding cell cycle progression. (**A**) OVCA429 and OVCA433 cells were transfected with control or PDE1A-specific siRNAs for 48 h. PDE1A protein expression was analyzed by western blotting (left), and intracellular cGMP levels were quantified (right). (**B**) Cell proliferation of OVCA429 (left) and OVCA433 (right) measured by EZ-Cytox assay after transfection with control siRNA or PDE1A siRNAs. Curves represent control (solid line with closed squares), siPDE1A#1 (dotted line with closed circles), and siPDE1A#2 (solid line with open triangles). Absorbance at 450 nm was measured at the indicated time points. Data are shown as mean ± SD of three independent experiments. (**C**) Clonogenic growth of OVCA429 and OVCA433 cells 14 days after transfection with control or PDE1A siRNAs. Left, representative images of crystal violet–stained colonies. Right, quantification of colony numbers (mean ± SD of three independent experiments). Scale bar: 1 cm. (**D**) Cell-cycle distribution of OVCA429 (upper panels) and OVCA433 (lower panels) analyzed by flow cytometry after staining with propidium iodide. Left, representative DNA content histograms. Right, percentage of cells in G1, S, and G2/M phases presented as bar graphs (mean ± SD of three independent experiments). The number of asterisks indicates statistical significance: **p < 0.05, ***p < 0.001. Error bars represent the mean ± SD of three independent experiments, each performed in triplicate

To investigate the functional role of PDE1A in ovarian cancer cell proliferation, we silenced its expression using siRNAs in two cell lines, OVCA429 and OVCA433, which exhibited relatively high endogenous PDE1A levels (Supplementary Fig. S1A). Knockdown efficiency was confirmed by qRT-PCR and western blotting (Supplementary Fig. S1B, Fig. 2A, left). Given that PDE1A is a Ca^2+^/calmodulin-dependent phosphodiesterase capable of hydrolyzing cGMP, we next examined the effects of PDE1A silencing on intracellular cGMP levels, measured using an ELISA-based Cyclic GMP XP assay as described in Methods (Section 2.15). PDE1A knockdown led to a significant increase in total intracellular cGMP levels in both OVCA429 and OVCA433 cells (Fig. 2A, right). Functionally, PDE1A silencing significantly reduced cell viability, as measured by the EZ-Cytox assay (Fig. 2B) and suppressed colony forming ability in both cell lines (Fig. 2C). To determine whether this growth inhibition was related to altered cell cycle progression, we performed flow cytometric analysis. PDE1A knockdown led to an accumulation of cells in the G1 phase, with a corresponding reduction in the S and G2/M phases (Fig. 2D), supporting the notion that PDE1A promotes G1–S cell-cycle transition in ovarian cancer cells.

### 3.3 PDE1A Regulates the Metastatic Ability of Ovarian Cancer Cells via the ***β***-Catenin Pathway

**Figure 3:**
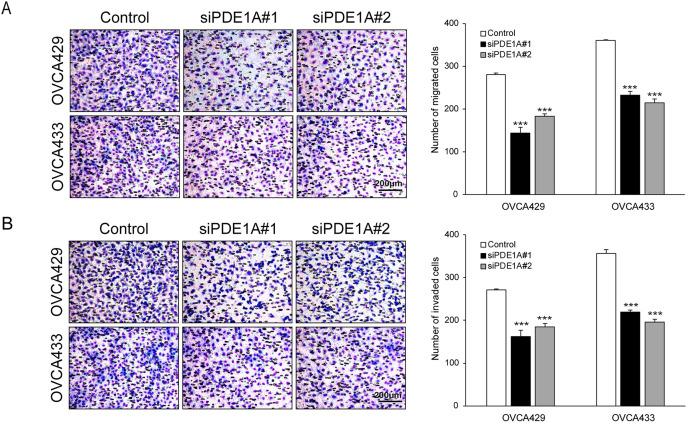

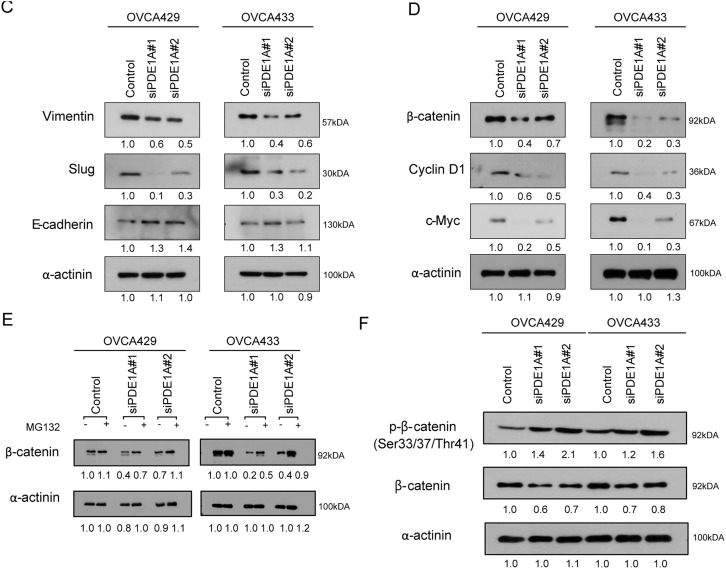
PDE1A regulates the metastatic ability of ovarian cancer cells through the β-catenin pathway. OVCA429 and OVCA433 cells were transfected with siRNAs against PDE1A for 48 h. (**A,B**) Cell migration and invasion assays were performed using Boyden chambers. Left panel: A representative image of the Boyden chamber assay. Right panel: Quantitative results of the Boyden chamber assay. Scale bar: 200 µm (**C,D**) Expression of vimentin, slug, E-cadherin, β-catenin, cyclin D1, c-Myc, and α-actinin was analyzed using the western blot analysis (numbers below each blot are densitometric values). (**E**) siRNA-transfected cells were incubated with DMSO or MG132 for 6 h. Expression of β-catenin and α-actinin was analyzed using the western blot analysis (numbers below each blot represent the densitometric values). (**F**) Phosphorylation of β-catenin at Ser33/Ser37/Thr41 was analyzed by western blotting following PDE1A knockdown. The numbers below each blot represent densitometric values. The number of asterisks indicates statistical significance: ***p < 0.001. Error bars represent Error bars represent mean ± SD of values from triplicate experiments

We next examined the effect of PDE1A on the migratory and invasive capacity of ovarian cancer cells. PDE1A knockdown significantly suppressed both migration and invasion by OVCA429 and OVCA433 cells (Fig. 3A,B). Migration was reduced by on average 41.7% in OVCA429 and 37.9% in OVCA433 cells, with similar inhibition of invasion. These consistent inhibitory effects across both cell lines support a role for PDE1A in promoting motility and invasiveness in EOC. We next assessed whether PDE1A modulates epithelial–mesenchymal transition (EMT). Western blot analysis revealed that PDE1A silencing reduced vimentin and Slug expression while increasing E-cadherin expression, consistent with EMT suppression (Fig. 3C). Because EMT can be driven, in part, by β-catenin activation, which is associated with invasion and metastasis in various cancers, we next investigated whether PDE1A influences β-catenin signaling. Indeed, β-catenin protein levels were markedly reduced in PDE1A-silenced cells (Fig. 3D). Notably, PDE1A-knockdown resulted in a substantial reduction in the levels of cyclin D1 and c-Myc, downstream transcriptional targets of β-catenin, whereas the mRNA level of CTNNB1, which encodes β-catenin, remained unchanged (Supplementary Fig. S2). The PDE1A-knockdown-induced reduction in β-catenin induced was abrogated by the proteasome inhibitor MG132, indicating that PDE1A knockdown promotes β-catenin degradation via a proteasome dependent mechanism (Fig. 3E). A key mechanism regulating β-catenin stability involves its phosphorylation by GSK3β: casein kinase I (CKI) first primes β-catenin by phosphorylating Ser45, which is followed by GSK3β-mediated phosphorylation at Ser33/Ser37/Thr41. These phosphorylation events mark β-catenin for recognition by the E3 ubiquitin ligase complex, leading to its ubiquitination and subsequent proteasomal degradation. To determine whether PDE1A modulates this regulatory cascade, we examined the phosphorylation status of β-catenin at Ser33/Ser37/Thr41 following PDE1A knockdown. Levels of β-catenin phosphorylated at Ser33/Ser37/Thr41 (p–β-catenin Ser33/Ser37/Thr41) were significantly elevated in the PDE1A-silenced cells (Fig. 3F). These findings are consistent with a model in which PDE1A enhances EMT and metastatic behavior in ovarian cancer cells, at least in part, by stabilizing β-catenin by limiting GSK3β-dependent phosphorylation and proteasomal degradation.

### 3.4 Knockdown of ***β***-Catenin Expression Inhibits the Proliferation, Migration, and Invasion of Ovarian Cancer Cells

**Figure 4:**
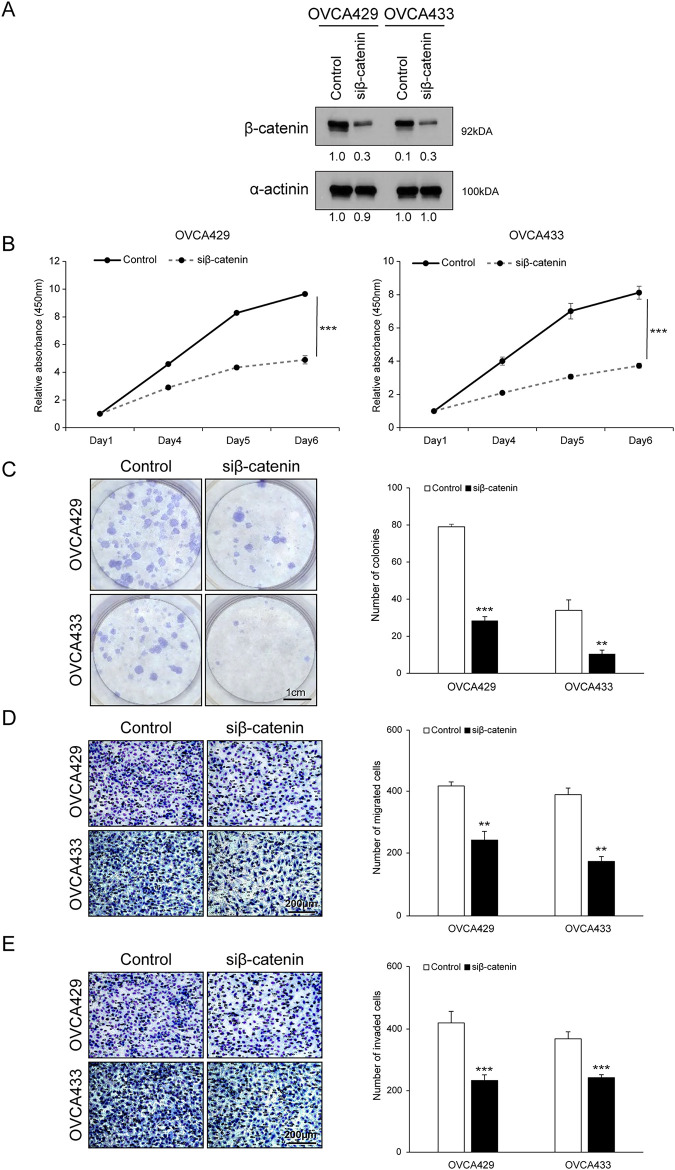
Knockdown of β-catenin expression inhibits the proliferation, migration, and invasion of ovarian cancer cells. OVCA429 and OVCA433 cells were transfected with an siRNA against β-catenin for 48 h. (**A**) Expression of β-catenin and α-actinin was analyzed using the western blot analysis. (**B**) Cell proliferation was detected using the EZ-Cytox assay (CCK-8–type), at the indicated time points. (**C**) Colony formation assay was performed 14 days after transfection. Left panel: Representative image of the colonogenic assay. Right panel: Quantitative results of the colonogenic assay. Scale bar: 1 cm (**D,E**) Cell migration and invasion assays were performed using the Boyden chamber assay. Left panel: A representative image of the Boyden chamber assay. Right panel: Quantitative results of the Boyden chamber assay. Scale bar: 200 µm. The number of asterisks indicates statistical significance: **p < 0.05, ***p < 0.001. Error bars represent mean ± SD of values from triplicate experiments

To directly assess the functional role of β-catenin in ovarian cancer cell proliferation and motility, we silenced its expression using siRNA in OVCA429 and OVCA433 cells. Efficient knockdown of β-catenin was confirmed by western blotting (Fig. 4A). β-catenin silencing resulted in a significant reduction in cell proliferation and colony formation (Fig. 4B,C) and significantly impaired the migratory and invasive abilities of both cell lines, as evidenced by the reduced Transwell membrane penetration (Fig. 4D,E). These findings suggest that β-catenin plays a key role in supporting the proliferative and metastatic potential of ovarian cancer cells.

### 3.5 LiCl Reversed the Inhibitory Effects of PDE1A Knockdown on Cell Proliferation, Migration, and Invasion

**Figure 5:**
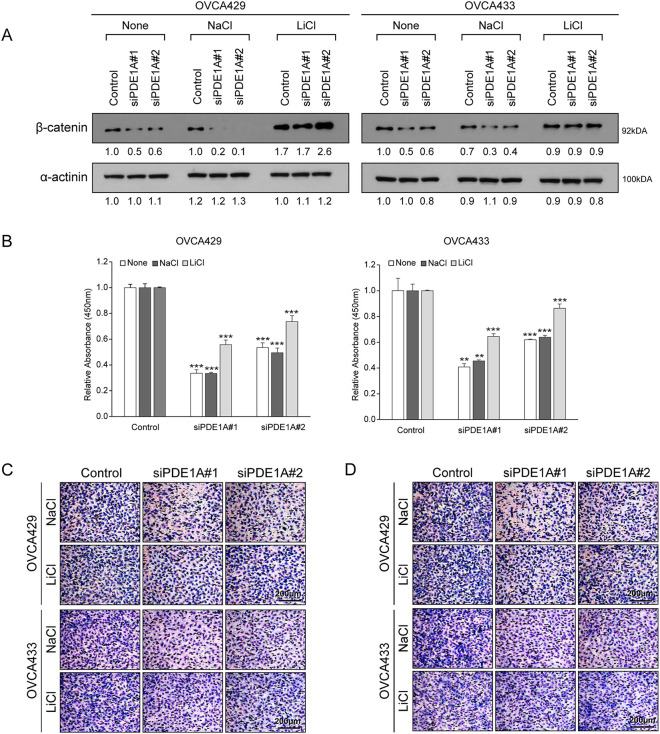

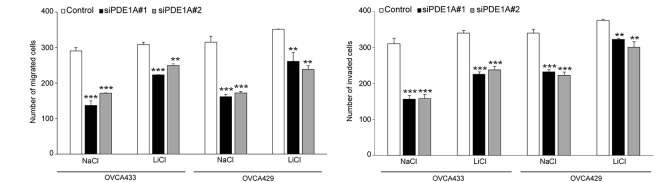
LiCl reversed the inhibitory effects of PDE1A knockdown on the proliferation, migration, and invasion of cells. OVCA429 and OVCA433 cells were transfected with siRNAs against PDE1A for 48 h and then treated with Wnt/β-catenin agonists (LiCl), or NaCl. (**A**) Expression of β-catenin and α-actinin was analyzed using the western blot analysis (numbers below each blot are densitometric values). (**B**) Cell proliferation was measured using the CCK-8 assay. (**C,D**) Cell migration and invasion assays were assessed using the Boyden chamber assay. Upper panel: A representative image of the Boyden chamber assay. Lower panel: Quantitative results of the Boyden chamber assay. Scale bar: 200 µm. The number of asterisk (*) indicates the level of significance: **p < 0.05, ***p < 0.005. Error bars represent mean ± SD of values from triplicate experiments

To further examine whether the growth-inhibitory effects of PDE1A knockdown are mediated through β-catenin, we performed rescue experiments using lithium chloride (LiCl), an activator of Wnt/β-catenin signaling [24]. OVCA429 and OVCA433 cells were transfected with PDE1A siRNAs and then treated with LiCl or control NaCl for 24 h. Western blot analysis demonstrated that LiCl treatment restored the β-catenin protein levels that were suppressed by PDE1A knockdown (Fig. 5A). Functionally, LiCl counteracted the inhibitory effects of PDE1A knockdown on cell proliferation (Fig. 5B), migration (Fig. 5C), and invasion (Fig. 5D). Together, these results support the view that β-catenin functions as a key downstream effector of PDE1A and that PDE1A contributes to ovarian cancer progression, at least in part, by sustaining β-catenin signaling activity. However, because LiCl was the only agent used to activate β-catenin in this study, potential effects on other signaling pathways cannot be completely excluded, and this should be considered a limitation of the present study.

## 4 Discussion

As a Ca^2+^/CaM-regulated phosphodiesterase, PDE1A controls cyclic nucleotide homeostasis and coordinates diverse pro-tumorigenic pathways. Although prior studies demonstrated its oncogenic activities in breast and bone cancers, the mechanistic role of PDE1A in ovarian cancer biology has not been comprehensively characterized [25]. PDE1A participates in promoting aggressive cancer phenotypes by modulating cell cycle progression and apoptosis, particularly in the basal subtype of breast cancer [26–28]. PDE1A expression is modulated by upstream CaMKK2, although this modulation exhibits histological subtype specificity in its correlation with survival outcomes [29]. Collectively, these reports implicate PDE1A as an oncogenic driver across multiple tumor types, yet its functional significance in EOC remains poorly defined as a gap that motivated the present investigation. Our data reveal strong associations between elevated PDE1A levels and unfavorable clinical parameters, specifically higher FIGO staging, increased histologic grade, and diminished platinum sensitivity. These observations position PDE1A as a tumor-promoting factor across multiple cancer types and highlight its candidacy as a novel prognostic biomarker for EOC [30–32]. Notably, other phosphodiesterase family members—including PDE3, PDE4, PDE5, and PDE10 isoforms—have also been implicated in ovarian cancer pathogenesis, underscoring the broader relevance of cyclic nucleotide signaling in this disease.

To further understand the clinicopathological role of PDE1A in patients with EOC, we explored its function in ovarian cancer cell lines, OVCA429 and OVCA433, through siRNA-mediated knockdown and assessed its effects on key cellular behaviors, such as proliferation, colony formation, migration, and invasion. Our findings demonstrate that reduction of PDE1A expression significantly inhibits these oncogenic activities in ovarian cancer cell lines and are in agreement with the findings of previous studies in which PDE1A was implicated in various malignancies, including breast cancer and osteosarcoma [29,33]. To elucidate the mechanisms underlying these phenotypic effects, we examined the influence of PDE1A knockdown on key signaling pathways. Our cGMP assays demonstrated that PDE1A knockdown increased intracellular cGMP in both OVCA429 and OVCA433 cells. Previous studies have shown that PDE1A can modulate cyclic nucleotide second messengers, including cAMP and cGMP, which are essential for cellular growth and survival. In particular, pharmacologic inhibition of PDE1A has been reported to enhance cGMP/PKG signaling and suppress Wnt/β-catenin activity in several cancer models, including EOC [24,34]. Our observation that PDE1A knockdown significantly reduced β-catenin levels and downregulated the expression of its downstream transcriptional targets, such as cyclin D1 and c-Myc, is consistent with this proposed cGMP/PKG–β-catenin axis. In addition, LiCl partially reversed the effects of PDE1A knockdown on β-catenin and its downstream targets, further supporting the involvement of Wnt/β-catenin signaling in PDE1A-driven EOC behavior. Together, our findings raise the possibility that inhibitors targeting the Wnt/β-catenin signaling pathway may be particularly relevant in PDE1A-overexpressing EOC, although this hypothesis requires further functional validation.

In phase I and II clinical trials, WNT974 (LGK974)—an orally available selective inhibitor of porcupine, a crucial Wnt ligand transporter—in combination with carboplatin induced cell cycle arrest in a higher percentage of ascites cells, in ascites samples from patients with primary EOC [35]. Similarly, Ipafricept (OMP54F28), another recombinant protein that inhibits Wnt signaling, was investigated in a phase IB study in combination with paclitaxel or carboplatin in patients with recurrent platinum-sensitive EOC [36]; that study revealed a median OS of 10.3 months, with an overall response rate of 75.7%, which is higher than that reported for historical data, such as that from the OCEANS (57%) and GOG 213 (56%) trials [36–38]. Here in this study, PDE1A knockdown induced G0/G1 phase arrest and reduced cyclin D1 expression. Together with previous reports that cGMP/PKG signaling can induce p21kip1-mediated growth arrest, these findings support the hypothesis that PDE1A may intersect with cGMP/PKG-dependent cell-cycle control, although we did not directly measure PKG activity or p21kip1 in this study. This aligns with the broader strategy of targeting cell cycle regulation in cancer therapy and suggests that further evaluation of PDE1A-directed approaches in EOC is warranted. More selective PDE1A inhibitors and *in vivo* EOC models will be required to clarify the therapeutic window and specificity of such strategies. Our findings thus provide evidence that PDE1A influences oncogenic signaling pathways, particularly the Wnt/β-catenin signaling pathway, supporting its potential as a viable therapeutic and prognostic biomarker for EOC. Further research is needed to deepen our understanding of its molecular mechanisms and to evaluate its clinical applicability in EOC.

We observed that PDE1A overexpression significantly modulated platinum-based chemotherapy resistance in EOC. Consistent with the potential of PDE inhibitors to enhance chemosensitivity, *Ribeiro and Vale* et al. reported that levosimendan, a calcium sensitizer with PDE3 inhibitory activity, exhibited synergistic cytotoxic effects when combined with 5-fluorouracil in bladder cancer models, highlighting its potential for drug repurposing in oncology [39]. In prostate cancer cells, co-treatment with sildenafil (a PDE5 inhibitor) and doxorubicin achieved synergistic effects, increasing surface localization of CD95 and concurrent inactivation of NF-κB, while also downregulating FLICE-like inhibitory protein (FLIP) and Fas associated phosphatase-1 (FAP-1); this suggests that PDE inhibitors can significantly augment chemotherapeutic efficacy by altering crucial cellular signaling pathways [40,41]. Collectively, these findings support the need for further exploration of the mechanism underlying the role of PDE1A in chemoresistance and suggest that PDE1A-targeted approaches should first be evaluated in preclinical combination models for platinum-resistant EOC, rather than being directly translated to clinical trial protocols.

Our study has several limitations that should be acknowledged. First, although we demonstrated that PDE1A knockdown increases intracellular cGMP and reduces β-catenin levels, we did not directly measure intracellular cAMP levels, PKG activity, or the protein expression of p21^Kip1. While our results align with the well-established cGMP/PKG signaling axis, direct quantification of these intermediates would strengthen the mechanistic link. Second, our functional experiments were restricted to *in vitro* cell line models; we did not utilize *in vivo* orthotopic or xenograft models, which are essential for evaluating the therapeutic window, toxicity, and systemic efficacy of PDE1A inhibition in a physiological context. Third, we focused exclusively on PDE1A and did not systematically investigate the potential compensatory upregulation of other phosphodiesterase families (e.g., PDE3, PDE4, or PDE5), which might attenuate the effects of PDE1A-specific targeting. Finally, the clinical component of our study was retrospective in nature, and large-scale prospective studies will be needed to validate the prognostic value of PDE1A in diverse EOC cohorts.

In summary, PDE1A overexpression marks epithelial ovarian cancers with advanced FIGO stage, poor tumor grade, reduced platinum sensitivity, and shorter disease-free and overall survival. Silencing PDE1A *in vitro* curtailed cell proliferation, colony formation, migration, and invasion, accompanied by β-catenin downregulation and G1 arrest; partial rescue by LiCl supports a PDE1A–β-catenin signaling axis. Overall, these findings support PDE1A as a prognostic biomarker and candidate therapeutic target in ovarian cancer. Given that phosphodiesterase inhibitors are already used clinically for other indications, repurposing or developing PDE1A-selective inhibitors—particularly in combination with platinum-based chemotherapy—warrants evaluation in larger patient cohorts and preclinical models

## Supplementary Materials



## Data Availability

The data that support the findings of this study are available from the Cor-responding Author, [Hanbyoul Cho], upon reasonable request.

## Supplementary Materials

The supplementary material is available online at https://www.techscience.com/doi/10.32604/or.2025.072105/s1.

## Abbreviations

ATCC: American Type Culture Collection
CaM: Calmodulin
cAMP: Cyclic adenosine monophosphate
cGMP: Cyclic guanosine monophosphate
DEG: Differentially expressed gene
DFS: Disease-free survival
DMEM: Dulbecco’s Modified Eagle Medium
EOC: Epithelial ovarian cancer
EMT: Epithelial–mesenchymal transition
FBS: Fetal bovine serum
FFPE: Formalin-fixed paraffin-embedded
FIGO: International Federation of Gynecology and Obstetrics
GEO: Gene Expression Omnibus
HCC: Hepatocellular carcinoma
iHOSE: Immortalized human ovarian surface epithelial
LiCl: Lithium chloride
OS: Overall survival
PDE1A: Phosphodiesterase 1A
PFS: Progression-free survival
qRT-PCR: Quantitative real-time polymerase chain reaction
RECIST: Response Evaluation Criteria in Solid Tumors
RNA: Ribonucleic acid
RNAiMAX: RNA interference MAX
TMA: Tissue microarray
WHO: World Health Organization
