# Supplementary material for: Clinical and Functional Characterization of PDE1A as a Wnt/β-Catenin-Linked Biomarker of Progression and Platinum Resistance in Epithelial Ovarian Cancer
Source: Oncol Res. 2026 Feb 24;34(3):16. doi: 10.32604/or.2025.072105 (PMC12963653; doi:10.32604/or.2025.072105)
Supplement: Supplementary file 1 [file OncolRes-34-72105-s001.docx]

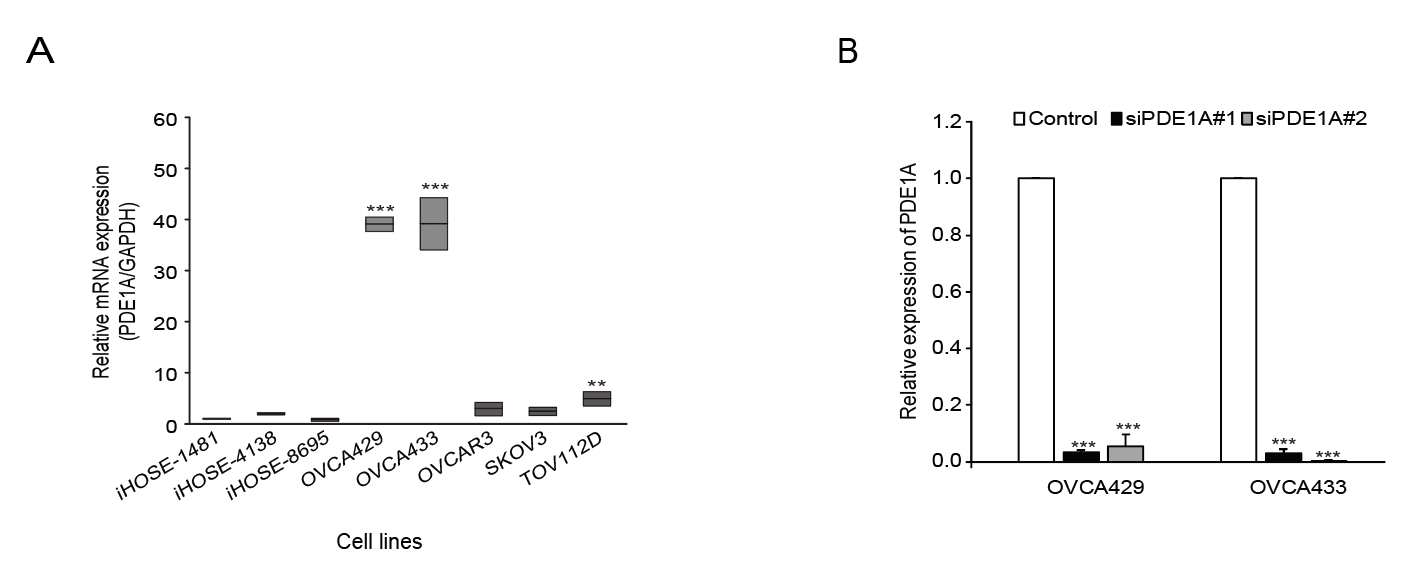


Supplementary Figure S1. Basal PDE1A expression and knockdown efficiency in ovarian cancer cell lines.

(A) Basal PDE1A mRNA expression in normal immortalized ovarian epithelial cells and ovarian cancer cell lines. OVCA429 and OVCA433 cells, which show relatively high endogenous PDE1A levels, were selected for functional assays. (B) Validation of PDE1A knockdown by siRNA in OVCA429 and OVCA433 cells assessed by quantitative reverse transcription polymerase chain reaction (qRT-PCR). The number of asterisks indicates statistical significance: ** *p* < 0.01, *** *p* < 0.001. Error bars represent the mean ± SD of three independent experiments, each performed in triplicate.


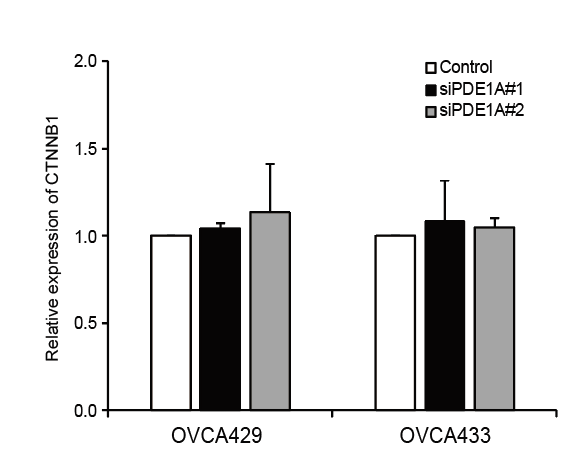


Supplementary Figure S2. CTNNB1 mRNA expression after PDE1A knockdown. Relative CTNNB1 mRNA levels in OVCA429 and OVCA433 cells transfected with control or PDE1A siRNAs for 48 h, measured by qRT-PCR. Error bars represent the mean ± SD of values from triplicate experiments.
